# Refining fine-mapping: Effect sizes and regional heritability

**DOI:** 10.1371/journal.pgen.1011480

**Published:** 2025-01-09

**Authors:** Christian Benner, Anubha Mahajan, Matti Pirinen

**Affiliations:** 1 Genentech, South San Francisco, California, United States of America; 2 Institute for Molecular Medicine Finland (FIMM), HiLIFE, University of Helsinki, Helsinki, Finland; 3 Department of Public Health, University of Helsinki, Helsinki, Finland; 4 Department of Mathematics and Statistics, University of Helsinki, Helsinki, Finland; The University of Chicago, UNITED STATES OF AMERICA

## Abstract

Recent statistical approaches have shown that the set of all available genetic variants explains considerably more phenotypic variance of complex traits and diseases than the individual variants that are robustly associated with these phenotypes. However, rapidly increasing sample sizes constantly improve detection and prioritization of individual variants driving the associations between genomic regions and phenotypes. Therefore, it is useful to routinely estimate how much phenotypic variance the detected variants explain for each region by taking into account the correlation structure of variants and the uncertainty in their causal status. Here we extend the FINEMAP software to estimate the effect sizes and regional heritability under the probabilistic model that assumes a handful of causal variants per region. Using the UK Biobank (UKB) data to simulate genomic regions, we demonstrate that FINEMAP provides higher precision and enables more detailed decomposition of regional heritability into individual variants than the variance component model implemented in BOLT or the fixed-effect model implemented in HESS, particularly when there are only a few causal variants in the fine-mapped region. Using data from 2,940 plasma proteins from the UKB study, we observed that on average FINEMAP identified 2.5 causal variants at an association signal and captured 36% more regional heritability than the variant with the lowest P-value. We estimate that in genomic regions with notable contribution to the total heritability, FINEMAP captures on average 13% and 40% more heritability than BOLT and HESS respectively. Our analysis shows how FINEMAP, BOLT and HESS relate to each other in cases where inference of a variant-level picture of the regional genetic architecture is possible.

## Author summary

Advancements in statistical methodologies to analyze Genome-Wide Association Studies (GWAS) have revealed that numerous genetic variants across the whole genome significantly contribute to the phenotypic variation in quantitative traits and disease risk. As sample sizes in GWAS grow, there is a continuous improvement in identifying and prioritizing individual variants that drive associations between genomic regions and phenotypes. To better understand both the individual and joint contribution of these variants to phenotypic variation, we extend the FINEMAP software to simultaneously estimate effect sizes of individual variants and heritability of genomic regions. Using simulations, we study how regional heritability estimates of FINEMAP and existing heritability approaches relate to each other and when a fine-mapping model is able to accurately narrow down the regional heritability into contributions from individual variants. In the analysis of plasma biomarkers, we demonstrate that FINEMAP offers more precise regional heritability estimates than existing heritability approaches in genomic regions that contribute notably to the total heritability. These new features in FINEMAP enable routine comparisons between the regional heritability estimates originating from different modeling assumptions, and inform us about the genetic architecture of each genomic region.

## Introduction

Over the last decade, GWAS have accumulated a massive catalog of robust statistical associations between numerous genomic regions and complex traits or diseases [1]. Typically, genetic variants highlighted by GWAS explain only a small fraction of total phenotypic variation (the missing heritability problem [2]) and are clustered into clumps of even hundreds of highly correlated variants giving rise to the fine-mapping problem [3].

The missing heritability problem has spurred highly successful methodological research in variance components models to evaluate the heritability contribution from the whole genome, not just from the statistically significantly associated variants [4–6]. Software packages implementing variance component models include EMMAX [7], GCTA [8], GEMMA [9], MMM [10] and BOLT [5]. Common to these implementations is the requirement of individual-level genotype-phenotype data and the assumption that the phenotype has a polygenic architecture under which all variants are causal with tiny effects. Heritability estimation using a polygenic model has also been carried out with LD Score regression [6] and a polygenic score method AVENGEME [11] from GWAS summary statistics without access to the original genotype-phenotype data. Together these methods have considerably narrowed the gap between estimated SNP heritability and heritability estimates from twin studies for many phenotypes and, importantly, shed light on the genetic architecture and enrichment of functional genetic variation in complex traits and diseases [12–13].

With increasing statistical power of GWAS comes a possibility to narrow the heritability down into specific regions of the genome. Heritability estimation in genomic regions from GWAS summary statistics was introduced in the software package HESS [14] under the assumption of arbitrary genetic architecture and a fixed-effect model for causal effect sizes. HESS estimates heritability through a regularized quadratic form built on marginal effect size estimates of all variants and their pairwise correlations to account for Linkage Disequilibrium (LD) between them. HESS was shown to be more accurate than LD Score regression in regional heritability estimation on simulated data when both methods were run with pairwise correlations either from the original genotype data or a reference panel.

Although polygenic and fixed-effect models have been very useful approaches for the estimation of genome-wide or regional heritability, the model assumptions of polygenicity or arbitrary genetic architecture likely leave room for improvement in accuracy if regional heritability can be attributed to only a few causal variants. Furthermore, our ultimate goal is to pinpoint the individual variants contributing to heritability and obtain accurate effect size estimates for them. Because existing heritability estimation methods do not provide such information, we turn to the fine-mapping framework instead.

The decisive solution to the fine-mapping problem is often hindered by the lack of information due to small effect sizes, high LD between variants and imperfect genotype information. Therefore, many recent fine-mapping methods [15–19] implement principled probabilistic quantification of causal variants that can then be used for downstream analyses. As GWAS sample sizes are soon counted in millions providing unprecedented accuracy for statistical fine-mapping, it would be useful to routinely evaluate how much of the regional heritability can be explained by the fine-mapped variants. In particular, we may expect that fine-mapping captures a large part of the regional heritability in 1) molecular endophenotypes with moderate to large effect sizes at causal variants, and 2) in large-scale biobank projects and ongoing GWAS meta-analyses with adequate statistical power to detect most individual causal variants even with smaller effect sizes. On the other hand, fine-mapping is likely not informative in regions with only tiny effect sizes or in GWAS with very small sample sizes. In cases where the fine-mapping seems to explain the total regional heritability, we have a much more informative picture of the regional architecture than is given by the existing heritability estimation methods that do not identify individual causal variants. Therefore, we have extended the FINEMAP software [18] to provide estimates of effect sizes and regional heritability. Our previously published FINEMAP algorithm (version 1.1) [18] worked only with small causal effect sizes and did not provide estimates of effect sizes or regional heritability. Here we introduce FINEMAP version 1.4 that estimates causal effects of arbitrary magnitude and estimates regional heritability by accounting for the uncertainty of the causal configuration and the LD-adjusted joint effect sizes of causal variants.

We compare regional heritability estimation using FINEMAP with both the variance component model implemented in BOLT and the fixed-effect model implemented in HESS. Ideally, with such comparisons, we can evaluate how much of the total regional heritability we capture with fine-mapped variants. Previously, Gusev et al. [20] have studied how the variance explained by the variant with the lowest P-value of a GWAS region compares to the results of a variance component model applied to the same region. They reported that on average the variance component model explained 1.29 times more heritability over nine diseases. We use plasma level concentrations of 2,940 unique proteins from the UKB, with a broad spectrum of genome-wide heritability levels. As an example where both FINEMAP and BOLT give a similar estimate of about 41% regional heritability, we study in detail the association between the *PON1* gene and Paraoxonase 1 levels. To interpret the results from our protein data analysis more generally, we also perform a simulation study to evaluate FINEMAP, BOLT and HESS under different genetic architectures. As our ultimate goal is to narrow down the polygenic regional heritability into individual variants, it is useful to know how the heritability estimates of FINEMAP, BOLT and HESS relate to each other in cases where such decomposition is indeed possible. More generally, an ability to do routine comparisons between the regional heritability estimates originating from different modeling assumptions, including a fine-mapping model, further informs us about the genetic architecture of each region.

## Results

We start with a motivating example of a region with a large contribution to the total heritability. Next, we study by simulation how a fine-mapping method FINEMAP, a variance component model BOLT and a fixed-effect heritability estimation model HESS perform more generally. Finally, we apply the methods to estimate the regional heritability of Protein Quantitative Trait Loci (pQTLs).

### Regional heritability of paraoxonase 1

Paraoxonase 1 (PON1) is a protein encoded by the *PON1* gene on chromosome 7. Serum levels of PON1 have been reported to be reversely associated with systemic oxidative stress and atherosclerosis risk [21]. The strongest genetic associations with the serum level of PON1 were indeed located near the *PON1* gene, which agrees with an earlier GWAS [21]. We applied FINEMAP on a 5.9Mb genomic region around the *PON1* gene with 1,095 variants. FINEMAP estimated that with over 95% probability 16 variants are needed to explain the association signal and the fine-mapped variants together explain a heritability of *h*^2^ = 0.42 (CI_0.95_: 0.40–0.43) which is considerably more than the lead variant rs854560 alone (*h*^2^ = 0.21, CI_0.95_: 0.20–0.22). The top configuration from FINEMAP included 16 variants which were compared to the top 16 variants identified by the stepwise conditioning (S1 Table). The log-likelihood difference was 48.2 in favor of the configuration identified by FINEMAP demonstrating the efficiency of the search algorithm of FINEMAP compared to the stepwise conditional analysis. BOLT estimated regional *h*^2^ = 0.41 (CI_0.95_: 0.39–0.44) which was similar to the estimate from FINEMAP but with a wider confidence interval. HESS with the default regularization gave an unexpectedly small value *h*^2^ = 0.33 (CI_0.95_: 0.32–0.34), that was larger than the marginal heritability contribution of the lead variant alone but clearly smaller than FINEMAP and BOLT. HESS without regularization of the LD matrix yielded a value *h*^2^ = 0.53 (CI_0.95_: 0.52–0.54) that was much larger than the values from FINEMAP and BOLT.

As a conclusion, the PON1 *cis-*pQTL is an example of a large effect region where FINEMAP provides fine-mapping that is statistically preferred over both the stepwise conditioning and single-variant analysis, and produces heritability estimates that are more detailed and more precise than the results from BOLT or HESS. However, paraoxonase 1 levels are an extreme case with a very large regional heritability contribution from the *cis*-pQTL and low polygenicity for the levels of PON1 outside of the *PON1* locus (BOLT *h*^2^ = 0.06, CI_0.95_: 0.02–0.10). Therefore, we next performed simulation studies to assess more generally the performance of FINEMAP, BOLT and HESS under different genetic architectures.

### Simulations with three causal variants in GWAS regions

Using datasets on 100 GWAS regions, we evaluated heritability estimation implemented in FINEMAP, HESS and BOLT in simulations with three randomly chosen causal variants. This setting of a very sparse genetic architecture is particularly suitable for FINEMAP and we are interested in how BOLT and HESS perform relative to FINEMAP in order to later interpret results from our protein data analysis.

**Fig 1:**
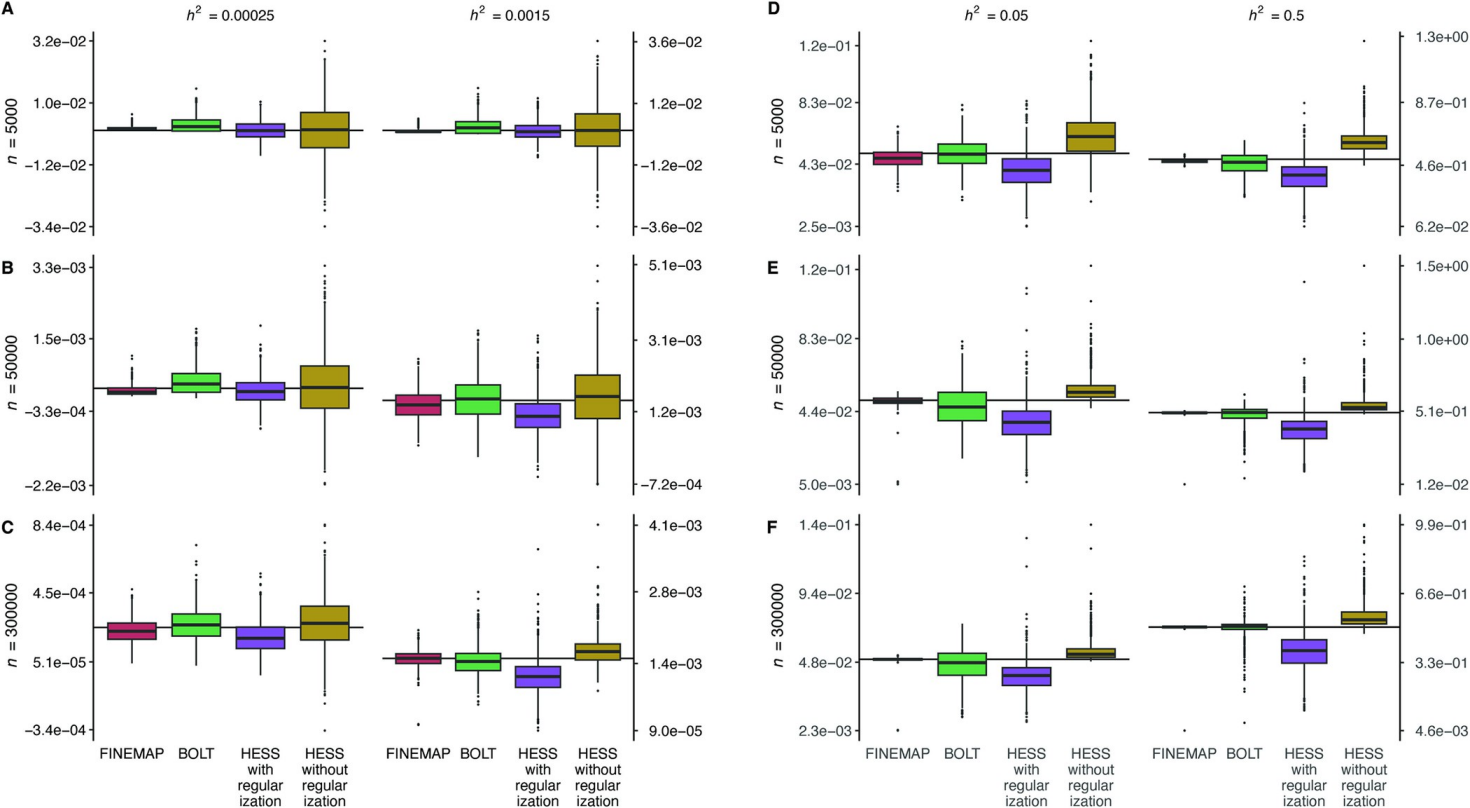
Heritability estimation on simulated data. Genotype data from UKB over 100 GWAS regions were used for phenotype generation. Three GWAS sample sizes (*n*) and four heritability values (*h*^2^) were considered and for each combination of region, sample size and heritability, 10 datasets were generated per GWAS region. Each dataset included three causal variants with joint effect sizes chosen so that the three variants together account for the regional heritability in proportions of 61.8%, 25.8% and 12.4%. For each of the three estimation methods, we show the boxplots of point estimates over the datasets. The horizontal lines mark the true value of heritability.

The FINEMAP results verified our implementation as the estimates were nearly unbiased and the precision increased with the sample size (Fig 1 and S2A Table). When the sample size was small or the heritability was low, FINEMAP tended to underestimate the heritability but this effect disappeared with increasing sample size. This phenomenon shows how our prior distributions favor sparse models and smaller effect sizes in cases where the information in the data is weak, but how the effect of the priors diminishes with more informative data. However, when the heritability is tiny and the data are uninformative (*n* = 5,000, *h*^2^ = 0.025%), FINEMAP can also overestimate the heritability (Fig 1A and S2A Table). BOLT was downwardly biased for the larger heritabilities but upwardly biased for the smaller heritabilities (Fig 1 and S2A Table). The downward bias for the large heritabilities may result from a clear violation of the polygenic model whereas the upward bias for the small heritabilities may exist because BOLT constrains the REML-estimates to reside in the interval [0,1] and hence can overestimate heritability values close to zero. Root mean square error (RMSE) of BOLT improved with increasing sample size except for a heritability value of 5%. Compared to FINEMAP, BOLT had larger RMSE (S2B Table). This suggests that the polygenic assumption of BOLT leads to less accurate estimates compared to FINEMAP in cases where heritability can be attributed to a few causal variants.

HESS with regularization indicated a systematic downward bias without improvement at larger sample sizes (Fig 1 and S2A Table). This may indicate that the regularization method applied by HESS can cause considerable underestimation of heritability in regions with only a few causal variants. We observed that HESS without regularization was upwardly biased across the sample sizes. The RMSE values from HESS were always larger than those from FINEMAP and, in most cases, also larger than those from BOLT (S2B Table).

To assess the calibration of uncertainty estimates of *h*^2^, we study the coverage of 95% credible and confidence intervals defined as the proportion of intervals that covered the true level of heritability (S2B Table). For FINEMAP, the coverage was always at least 86% while for some of the 95% intervals of BOLT the coverage dropped below 10%. For all sample sizes, the 95% intervals from HESS with and without regularization were clearly miscalibrated for the larger heritabilities because of the systematic bias in heritability estimates. S2B Table also shows the ratio between the average standard error (BOLT and HESS) or average posterior standard deviation (FINEMAP) and the standard deviation of the heritability estimates of the respective method. If the estimated standard error or posterior standard deviation was frequentistically well calibrated, we would expect this ratio to be close to one. We see that for FINEMAP and HESS without regularization, in most scenarios, the ratio is in the range of 0.8–1.2 showing a reasonably good frequentist calibration. For BOLT and HESS with regularization the ratio is systematically above one and often clearly above 1.2, which indicates a worse frequentist calibration of the standard error estimates.

S1A Fig shows a comparison of marginal posterior inclusion probabilities (PIPs) between FINEMAP and CAVIARBF across datasets with three causal variants. The strategies for handling large effect regions yield similar PIPs for both methods, but we note that CAVIARBF does not produce estimates of effect sizes or regional heritability. S1B Fig shows a comparison of PIPs from FINEMAP using different strategies to model large effect regions. FINEMAP v1.4 performs well in all settings whereas we observe many false positives when large effects are not appropriately accounted for in the previously published FINEMAP v1.1. This is because when we incorrectly assume that all effects are small, the only way to explain large regional heritability is by stipulating that also many non-causal variants contribute to the heritability.

### Simulations with fifty causal variants in GWAS regions

We also evaluated FINEMAP, BOLT and HESS in simulations with fifty randomly chosen causal variants using the 100 GWAS regions. This setting is less suitable for FINEMAP than the previous setting with three causal variants because individual causal variants now explain much less heritability. However, as large biobank data can provide adequate statistical power to detect many individual causal variants even with small effect sizes, these results are useful when we interpret the results from our protein data analysis.

**Fig 2:**
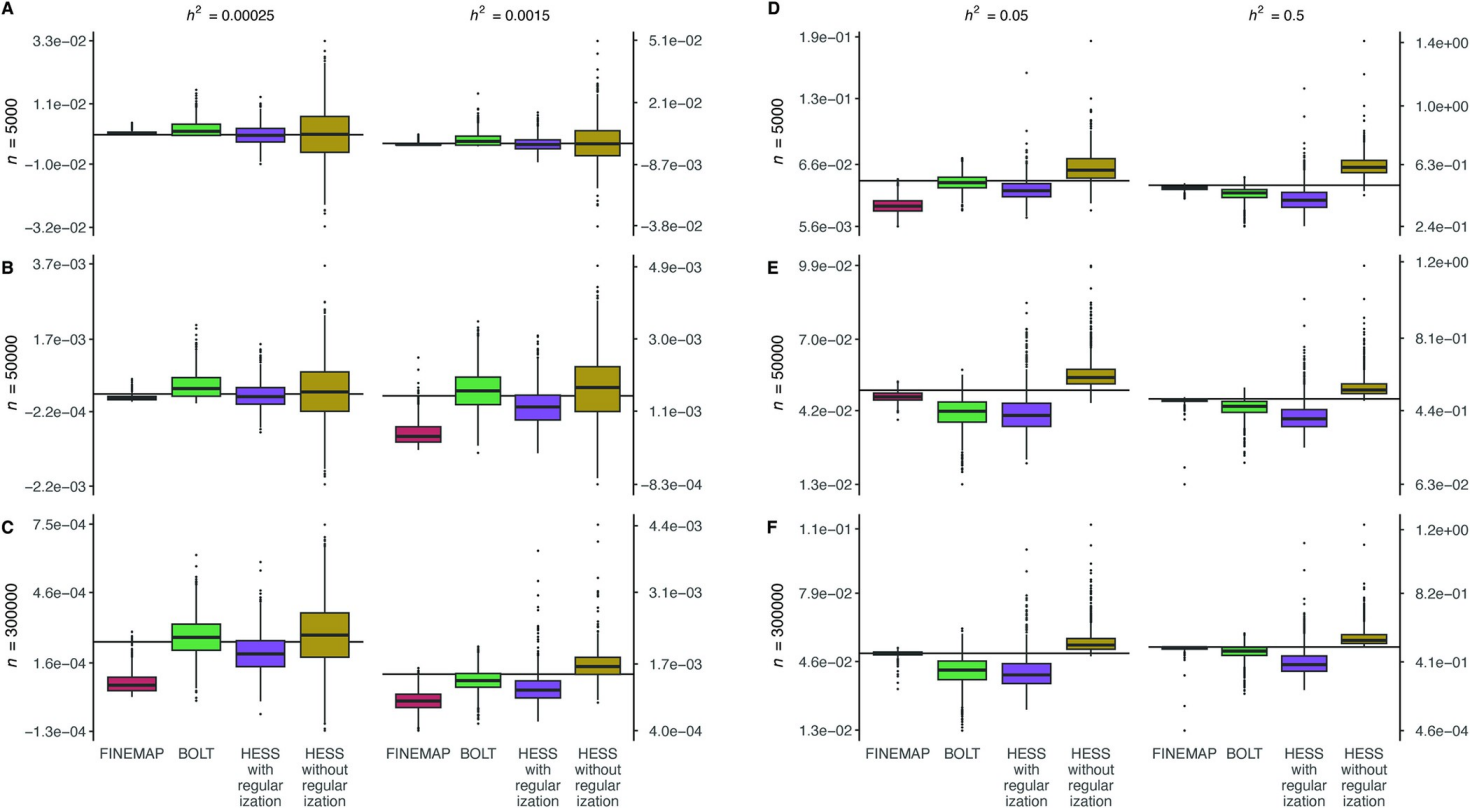
Heritability estimation on simulated data. Genotype data from UKB over 100 GWAS regions were used for phenotype generation. Three GWAS sample sizes (*n*) and four regional heritability values (*h*^2^) were considered and for each combination of region, sample size and heritability, 10 datasets were generated per GWAS region. Each dataset included 50 causal variants with equal effect size. For each of the three estimation methods, we show the boxplots of point estimates over the datasets. The horizontal lines mark the true value of heritability.

FINEMAP was downwardly biased for the smallest *h*^2^ of 0.025% and 0.15% (Fig 2A–2C) for all sample sizes except for the smallest sample size *n* = 5,000 when *h*^2^ was 0.025% (Fig 2A). For larger *h*^2^ of 5% and 50%, FINEMAP underestimated the heritability when *n* = 5,000 but this effect diminished with increasing sample size. These results show how the fine-mapping priors, that favor sparse causal models, tend to underestimate the heritability when the heritability is scattered across numerous tiny effects but this underestimation decreases when both the effect sizes and the sample sizes increase. BOLT was weakly upwardly biased for the smallest *h*^2^ of 0.025% and 0.15% except when *h*^2^ was 0.15% and *n* = 300,000 (Fig 2A–2C). For larger heritabilities, BOLT provided downwardly biased heritability estimates (Fig 2D–2F). Heritability estimates from HESS in simulations with fifty causal variants were similar to results in simulations with three causal variants (S2A, S2B, S3A and S3B Tables).

For FINEMAP, the coverage (S3B Table) was below 30% for all sample sizes and heritabilities except for the smallest *h*^2^ of 0.025% when *n* = 5,000 and *n* = 50,000 as well as for *h*^2^ of 0.15% and *n* = 5,000 when coverage was above 84%. The coverage of BOLT was always above 72%, except at *h*^2^ of 0.025% when *n* = 5,000 and at *h*^2^ of 50% when *n* = 300,000 the coverage dropped below 60%. For all sample sizes, the 95% intervals from HESS with and without regularization were clearly miscalibrated for the larger heritabilities because of the systematic bias in heritability estimates. For FINEMAP, BOLT and HESS with regularization the ratio between the average standard error (BOLT and HESS) or average posterior standard deviation (FINEMAP) and the standard deviation of the heritability estimates of the respective method was systematically above one and often clearly above 1.2, which indicates a worse frequentist calibration of the standard error estimates. For HESS without regularization the ratio is in the range of 0.8–1.0 showing a reasonably good frequentist calibration.

We observed that as heritability and sample size increase, the expected number of causal variants estimated by FINEMAP becomes a reliable statistic to inform us about whether the genetic architecture in the region is sparse or polygenic. In S2 Fig we see that this statistic makes a clear distinction between the sparse and polygenic architectures starting from the setting when *h*^2^ = 0.15% and *n* = 300,000.

### Protein-associated genomics regions

**Fig 3:**
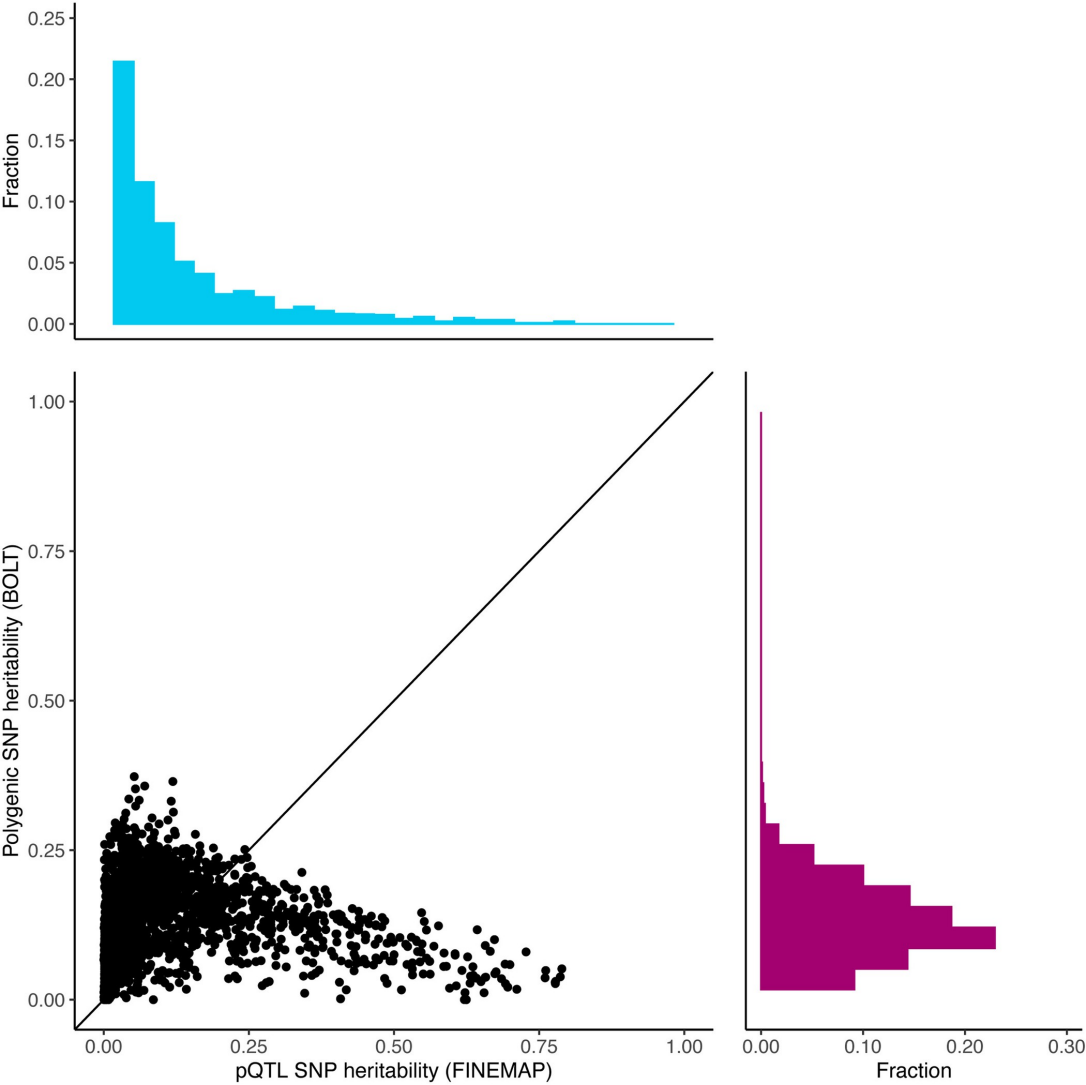
Heritability estimation on data from 2,416 unique proteins. Each circle represents the point estimate of heritability (*h*^2^) for the pQTL component (sum of variance explained across all pQTLs) vs. the polygenic component (genome-wide heritability excluding regions defined by pQTLs ±5Mb). Heritability was estimated with FINEMAP and BOLT using up to 28,008 unrelated individuals of European ancestry from UKB and genotyped biallelic variants with MAF greater than 1%.

**Fig 4:**
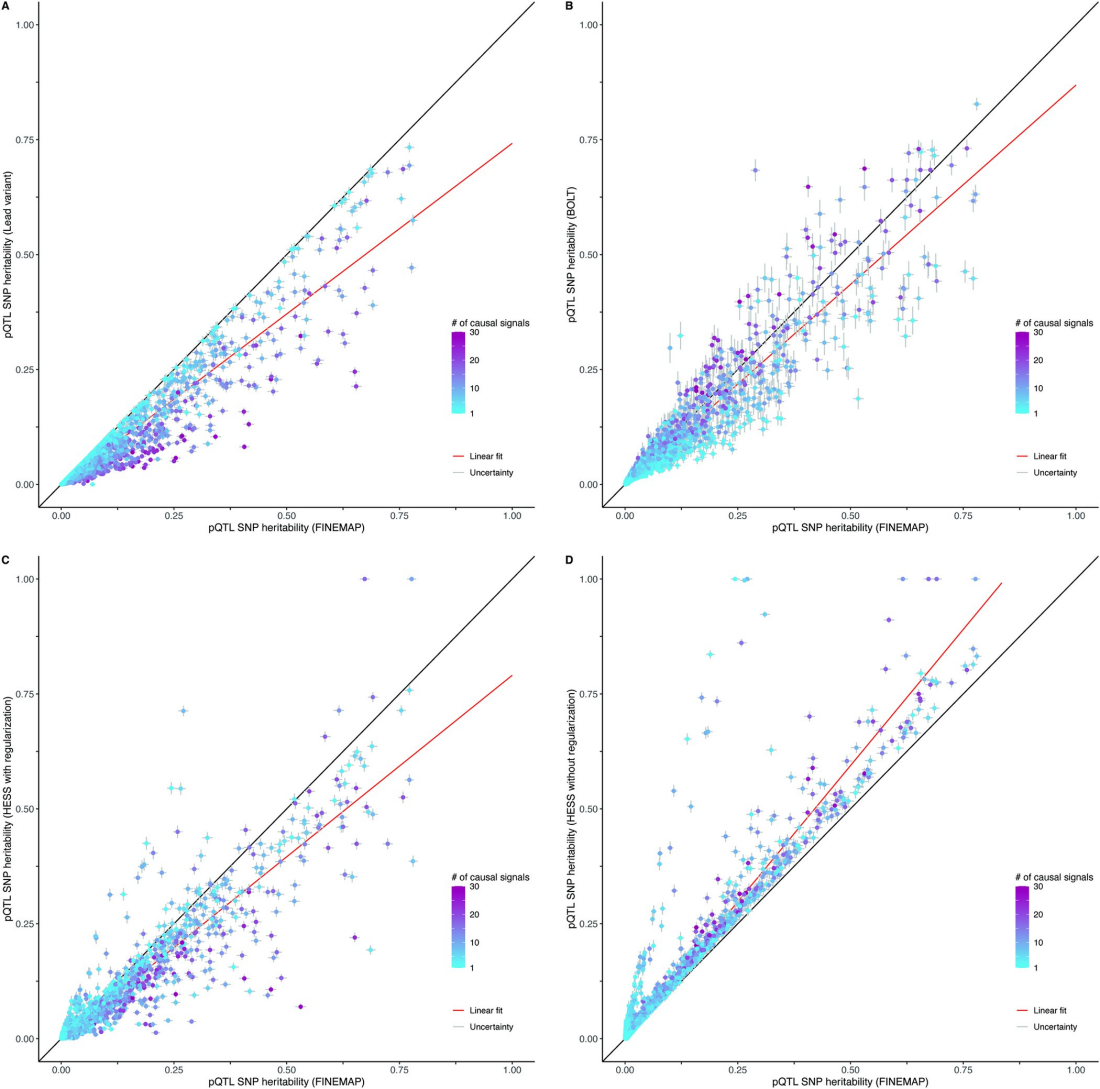
Heritability estimation on data from 2,490 unique proteins. Point estimates of heritability (*h*^2^) are shown for 14,189 pQTLs together with their estimated 95% uncertainty intervals. Regional heritability was estimated in up to 28,008 unrelated individuals of European ancestry from UKB using genotyped biallelic variants with MAF greater than 1%. Panels compare estimates from FINEMAP with the estimates from the lead variant alone (A), BOLT (B) or HESS (C-D). The posterior expected number of causal variants is estimated by FINEMAP and highlighted by color gradient.

GWAS of 2,490 unique proteins identified 14,189 genome-wide significant pQTL regions. Using BOLT, we estimated an average polygenic component of heritability from outside the pQTL regions of 0.11 per protein ranging from 0.00 (Transgelin-3, UniProt: Q9UI15) to 0.37 (Cystatin-M, UniProt: Q15828), whereas in 23% of the proteins causal variants in pQTLs estimated by FINEMAP explained more protein level variation than the polygenic component (Fig 3) and on average 36% more regional heritability than the lead variant alone (Fig 4A). FINEMAP analyses further showed that the posterior expected number of causal variants is 2.5 on average, ranging from 1 (Uromodulin, UniProt: P07911) to 30 (Amnionless, UniProt: Q9BXJ7), and that the minor allele frequency (MAF) of causal variants is 0.24 on average, ranging from 0.02 (ADAMTS-like protein 4, UniProt: Q6UY14) to 0.47 (Carcinoembryonic antigen-related cell adhesion molecule 5, UniProt: P06731). The region-specific results are given in S4 Table.

**Fig 5:**
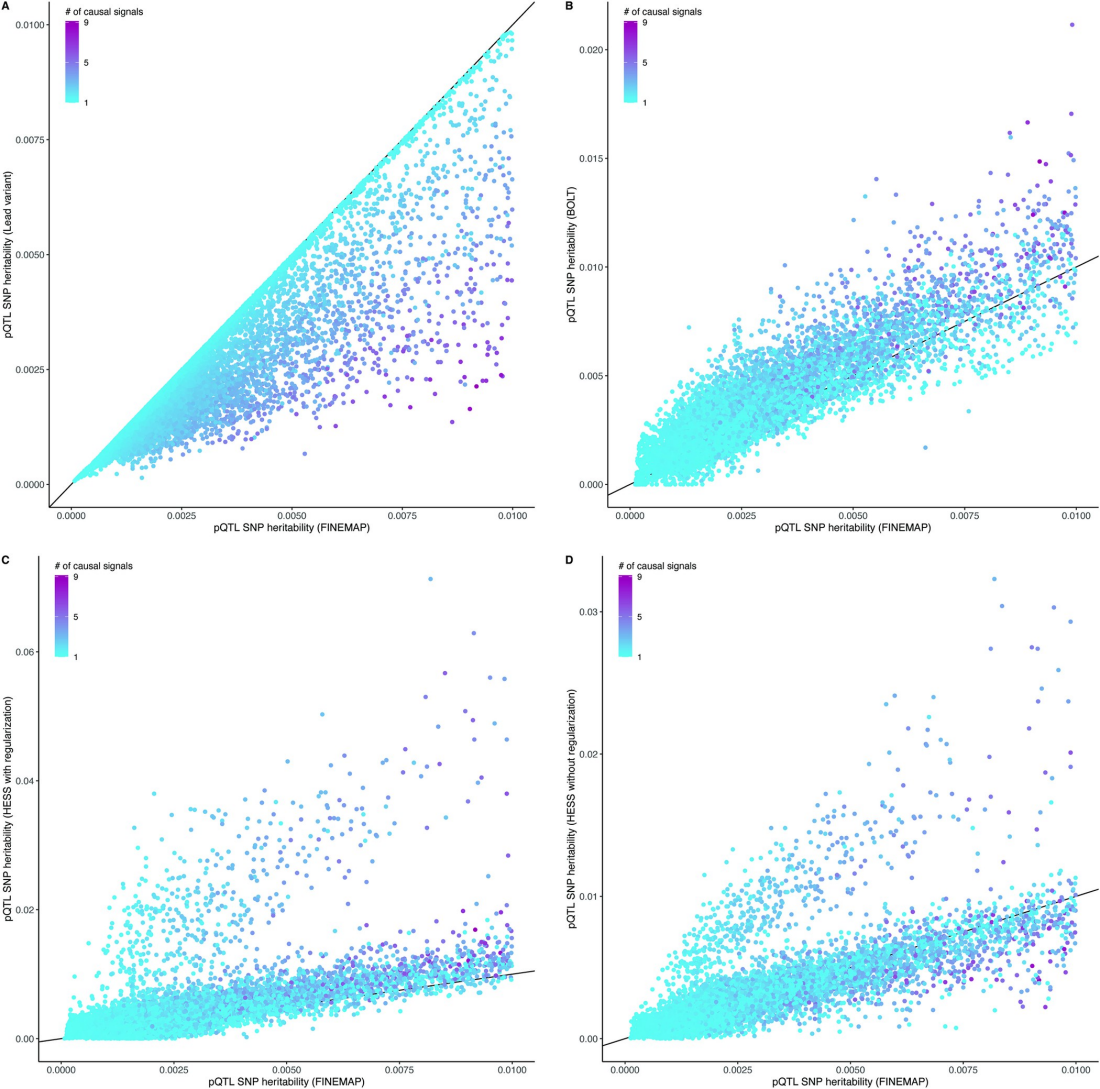
Heritability estimation on data from 2,490 unique proteins when FINEMAP estimate is δ 0.01. Point estimates of heritability (*h*^2^) are shown for 11,887 pQTLs together with their estimated 95% uncertainty intervals. Regional heritability was estimated in up to 28,008 unrelated individuals of European ancestry from UKB using genotyped biallelic variants with MAF greater than 1%. Panels compare estimates from FINEMAP that are less or equal than 0.01 with the estimates from the lead variant alone (A), BOLT (B) or HESS (C-D). The posterior expected number of causal variants is estimated by FINEMAP and highlighted by color gradient.

We further evaluated whether polygenic or fixed-effects modeling implemented in BOLT and HESS, respectively, detect an excess of heritability in the pQTL regions not captured by the FINEMAP model. When FINEMAP estimated regional heritability above *h*^2^ = 0.01 (2,302 / 14,189 regions), we observed 6.28 (CI_0.95_: 1.24–18.70) causal variants on average and the heritability estimates from FINEMAP were on average 13% larger than BOLT and 40% larger than HESS with default regularization (Fig 4B and 4C). This suggests that the regional genetic architecture of these pQTLs is not highly polygenic. For the regions where FINEMAP estimated a heritability level below 0.0l, the estimates from FINEMAP were on average 14% lower than BOLT and HESS with default regularization (Fig 5B and 5C). Compared to FINEMAP, the heritability estimates from HESS without regularization (Figs 4D and 5D) were 57% larger on average.

BOLT and HESS with default regularization provided, respectively, 97% and 178% wider 95% confidence intervals on average compared to the 95% credible intervals from FINEMAP when the estimated regional heritability by FINEMAP was less than *h*^2^ = 0.01. When the heritability captured by FINEMAP was more than *h*^2^ = 0.01, BOLT and HESS with default regularization provided respectively 83% and 20% wider 95% confidence intervals on average compared to FINEMAP. HESS without regularization provided 95% confidence intervals that were 162% wider on average compared to 95% credible intervals from FINEMAP.

## Discussion

Large biobanks with hundreds of thousands of samples are boosting our ability to fine-map genomic regions, that is, to probabilistically quantify which of the many correlated genetic variants have a putative causal effect on complex traits and diseases. As the power to identify causal variants and the precision of their effect size estimates increases, it is useful to be able to routinely evaluate how much of the phenotypic variance these causal variants explain together and how that compares to regional heritability estimates from models with different assumptions about the genetic architecture. To our knowledge, an estimate of the regional heritability of the fine-mapped variants is not given by the existing probabilistic fine-mapping methods. In this work, we extended the FINEMAP software to output estimates of effect sizes and regional heritability by taking into account the LD structure among the variants and the posterior probability of each putative causal configuration of variants.

We performed simulations to verify our implementation and to assess how existing heritability estimation methods BOLT and HESS work in the case where a genomic region truly contains only a few causal variants. Previously, BOLT and HESS have been tested under highly polygenic architectures and here we assessed how they perform when there is considerable regional heritability but its source is not polygenic. We see this as an important piece of information since our goal is to interpret what the possible differences in the regional heritability estimates from FINEMAP, BOLT and HESS reveal about the genetic architecture of the region. We also considered highly polygenic settings where genetic effects would be distributed among numerous variants with tiny effects. In such settings, we expect that reduced statistical power to detect individual causal variants makes fine-mapping difficult and consequently also keeps the regional heritability estimate of FINEMAP low, which was confirmed by our results. Based on previous analyses [14], we expected that some existing methods, such as BOLT and HESS, could still provide reasonable heritability in such highly polygenic regions.

Our simulation results show that FINEMAP is nearly unbiased for larger sample sizes and provides accurate uncertainty quantification of the regional heritability estimates when there are only a few causal variants in the region. With BOLT, there was an upward bias for the smallest heritability values possibly because BOLT constrains the REML-estimates to be positive. BOLT overestimated the standard error and had wider confidence intervals compared to FINEMAP, likely due to the simulation setup which differed from the polygenic modeling assumptions of BOLT and was similar to the FINEMAP model.

In simulations with 50 causal variants per region, we observed that FINEMAP underestimated the heritability, especially when both the regional heritability and the sample size were small. This is expected since the fine-mapping model assumes sparse genetic architecture, and therefore is not expected to perform well when there are as many as 50 causal variants with small effects. In this setting, BOLT typically provided smaller mean square error and better calibration of 95% intervals than FINEMAP.

We also observed that the default regularization method applied by HESS induces a systematic downward bias and low precision in the estimates. The default regularization corresponds to using only the first 50 principal components of the correlation structure of the variants to remove noise present in the trailing components, especially when the LD structure does not match the GWAS summary statistics. A possible explanation for the downward bias is that the weight of the variants contributing to heritability may be weak on the leading principal components and therefore using the default settings in HESS misses important information. In our simulations, we had a perfect match between the LD structure and GWAS summary statistics and we observed that by deactivating the regularization method we could remove the bias for the smallest heritability levels. However, this could not solve the problem for the largest heritability level and led to larger confidence intervals that increased with GWAS sample size. In the protein data analysis, we also observed that deactivating regularization of HESS does not always work even when we have access to the original LD structure.

Based on our simulations, our expectation in the analysis of protein data was that the FINEMAP heritability estimates would be bounded from below by the contribution from the variant with the lowest P-value alone and from above by polygenic and fixed-effect models, such as BOLT and HESS, that conceptually consider all variants causal. We indeed observed such a pattern with FINEMAP explaining 36% more regional heritability on average than the variant with the lowest P-value alone. The regional heritability estimates from BOLT tended to be lower than the values from FINEMAP in 58% of pQTLs (977 / 2,302 regions) in which FINEMAP estimated a heritability greater than 0.01 (2,302 / 14,182 regions). A possible explanation is that these pQTLs may contain a heritability contribution that is not highly polygenic and fine-mapping accurately captures it with our sample size of 28,008 individuals. In 78% of pQTLs (9,313 / 11,880 regions) in which regional heritability estimates from FINEMAP were less than 0.01 (11,880 / 14,182 regions), BOLT regional heritability estimates were larger compared to FINEMAP. Similar to our simulations, a part of the higher point estimates of BOLT may result from BOLT constraining the REML-estimates to reside in the interval [0,1] and hence overestimating heritability values close to zero and/or FINEMAP not capturing the lower heritability levels in these pQTLs. The estimates from HESS with default regularization were on average 5% larger compared to FINEMAP. Deactivation of the regularization method of HESS increased the HESS estimates so that they became 57% larger than the values from FINEMAP. A possible explanation is the presence of (nearly) perfectly correlated variants in the pQTL regions making the correlation matrices not invertible or unstable in genomic regions with (nearly) perfectly correlated variants.

An ultimate goal in genetics research is to narrow down the polygenic regional heritability into individual variants contributing to heritability and to obtain accurate effect sizes for them. The extensions for effect size and heritability estimation that we have introduced in the FINEMAP software provide a computationally efficient framework to deduce a variant-level picture of the regional genetic architecture. We expect that these new features prove useful as the accuracy of fine-mapping constantly increases with rapidly growing sample size of biobank-scale studies.

## Materials and methods

### Ethics statement

We used data from the UKB [22] using the Application number 44257. Ethics approval for the UKB study was obtained from the North West Centre for Research Ethics Committee (11/ NW/0382). Proteomic profiling of the UKB was approved by the Access Subcommittee of UKB, under Access Management System Application No. 65851. All participants of the UKB provided written informed consent.

### FINEMAP model

The goal of statistical fine-mapping is to refine a large set of genetic variants simply associated with a phenotype down to a much smaller set of variants with direct effect on the phenotype by accounting for the complex correlations between the variants. Although we call the variants with such direct effects “causal”, we bear in mind that further laboratory experiments would be required to establish the exact biological mechanisms of those variants.

The statistical framework in FINEMAP (see Web Resources) to infer causal variants is built on Bayesian variable selection for linear regression models [23] using Bayes factors. Previously [18], we derived the Bayes factor to assess the support for each combination of *k* variants denoted by *γ*, and hereafter called causal configuration, over the null configuration. Our previous derivation^18^ implemented in FINEMAP v.1.1, started with a likelihood approximation for the joint linear regression model for all variants in the region and assumed small enough effect sizes that we could ignore the differences in residual variance between causal configurations. In the following, we derive a corresponding Bayes factor by using a likelihood approximation of only the causal variants, which makes the effect size estimation feasible, and by allowing different residual variances for different configurations. For this, we assume the following Bayesian linear regression model for the causal configuration *γ*,

$$\begin{array}{l} y|\lambda_{\gamma }{,X}_{\gamma },{\hat{\sigma }}_{\gamma }^{2}∼N({y|X}_{\gamma }\lambda_{\gamma },{\hat{\sigma }}_{\gamma }^{2}I_{n})\\ \;\lambda_{\gamma }|s_{\lambda }^{2}∼N(\lambda_{\gamma }|0,s_{\lambda }^{2}I_{k}) \end{array}$$ (1)

where **y** is a standardized vector with phenotypic values for *n* individuals (with $\hat{E}(y)=0,\;\hat{Var}(y)=1),\;X_{\gamma }$ is a matrix of dimension *n*×*k* with standardized genotypes at the variants, ***λ***_*γ*_ are the additive effect sizes of the standardized variants, $s_{\lambda }^{2}$ is the user-given prior variance for the effect sizes and ${\hat{\sigma }}_{\gamma }^{2}$ is the point estimate of the residual variance in the multiple linear regression that we learn from the data (details below). Integrating out effect sizes ***λ***_*γ*_ analytically yields the marginal likelihood of the causal configuration

$$\begin{array}{l} p({y|X}_{\gamma },{\hat{\sigma }}_{\gamma }^{2},s_{\lambda }^{2})=\int N({y|X}_{\gamma }\lambda_{\gamma },{\hat{\sigma }}_{\gamma }^{2}I_{n})N(\lambda_{\gamma }|0,s_{\lambda }^{2}I_{k})d\lambda_{\gamma }\\ \;=N(y|0,{\hat{\sigma }}_{\gamma }^{2}I_{n}+s_{\lambda }^{2}X_{\gamma }X_{\gamma }^{T})\\ \;\propto {\left({\hat{\sigma }}_{\gamma }^{2}\right)}^{-n/2}{det(I_{k}+s_{\lambda }^{2}/{\hat{\sigma }}_{\gamma }^{2}X_{\gamma }^{T}X_{\gamma })}^{-1/2}\\ \;\times exp\left\{-\frac{1}{2{\hat{\sigma }}_{\gamma }^{2}}(y^{T}y-y^{T}X_{\gamma }{\left({\hat{\sigma }}_{\gamma }^{2}/s_{\lambda }^{2}I_{k}+X_{\gamma }^{T}X_{\gamma }\right)}^{-1}X_{\gamma }^{T}y)\right\} \end{array}$$

where we reduced computational complexity by applying the Matrix determinant lemma and Woodbury matrix identity on line 2. The computational complexity of evaluating the marginal likelihood can be further reduced substantially by replacing the phenotype-genotype data with GWAS summary statistics and pairwise correlations between variants [15–19]. That is, we replace individual-level data (***y***,***X***) with GWAS summary data $\left(\hat{z},\hat{R}\right)$ in which case

$$\begin{array}{l} \;X_{\gamma }^{T}y=\sqrt{n}{\hat{D}}_{\gamma }^{\frac{1}{2}}{\hat{z}}_{\gamma }\\ X_{\gamma }^{T}X_{\gamma }=n{\hat{R}}_{\gamma } \end{array}$$

where ${\hat{z}}_{\gamma }$ is a vector of ratios of effect size estimates and their standard errors from individual univariate linear regressions of each variant in the causal configuration, ${\hat{R}}_{\gamma }$ is a matrix of pairwise sample correlations among the variants and $\hat{D}$ is a diagonal matrix of estimates for residual variances in the univariate linear regressions. Thus, we can compute the Bayes factor to assess the support for a causal configuration *γ* over the null configuration *γ*_0_, that contains no variants, by using only GWAS summary data on variants included in the causal configuration *γ*

$$\begin{array}{l} BF(\gamma :\gamma_{0})=\frac{p(y|X_{\gamma },{\hat{\sigma }}_{\gamma }^{2},s_{\lambda }^{2})}{p({y|X}_{0})}\\ \;={\left({\hat{\sigma }}_{\gamma }^{2}\right)}^{-n/2}det{\left(I_{k}+{{ns}_{\lambda }^{2}/\hat{\sigma }}_{\gamma }^{2}{\hat{R}}_{\gamma }\right)}^{-1/2}\\ \;\times exp\left\{\frac{n}{2}-\frac{n}{2{\hat{\sigma }}_{\gamma }^{2}}(1-{\hat{z}}_{\gamma }^{T}{\hat{D}}_{\gamma }^{1/2}{\left({{\hat{\sigma }}_{\gamma }^{2}/s}_{\lambda }^{2}I_{k}+n{\hat{R}}_{\gamma }\right)}^{-1}{\hat{D}}_{\gamma }^{1/2}{\hat{z}}_{\gamma })\right\} \end{array}$$

Numerically, this Bayes factor agrees with our earlier derivation [18] when causal variants have small effect sizes and hence ${\hat{\sigma }}_{\gamma }^{2}\approx 1$. With the current derivation of the Bayes factor, we can also account for causal variants with larger effect sizes by allowing for different residual variances for different configurations and we provide a simple way to extract the posterior distribution of the effect sizes as explained next.

### Estimation of effect sizes and regional heritability

The posterior distribution of the effect sizes of variants included in a causal configuration is

$$\lambda_{\gamma }|y,X_{\gamma },{\hat{\sigma }}_{\gamma }^{2},s_{\lambda }^{2}∼N(\lambda_{\gamma }|\mu_{\gamma },\Sigma_{\gamma })$$

where $\Sigma_{\gamma }={\left(s_{\lambda }^{-2}I_{k}+n{\hat{\sigma }}_{\gamma }^{-2}{\hat{R}}_{\gamma }\right)}^{-1}$ and $\mu_{\gamma }=\sqrt{n}{\hat{\sigma }}_{\gamma }^{-2}\Sigma_{\gamma }{\hat{D}}_{\gamma }^{1/2}{\hat{z}}_{\gamma }$. An estimate of the heritability of a causal configuration *γ* is given by the ratio between the variance explained by the causal variants and the total variance of the phenotype, which we here compute conditional on the effect sizes ***λ***_*γ*_ as

$${\hat{h}}_{\gamma }^{2}=\frac{\hat{Var}(X_{\gamma }\lambda_{\gamma })}{\hat{Var}(y)}=\hat{E}(\lambda_{\gamma }^{T}X_{\gamma }^{T}X_{\gamma }\lambda_{\gamma })-\hat{E}{\left(X_{\gamma }\lambda_{\gamma }\right)}^{2}=\lambda_{\gamma }^{T}{\hat{R}}_{\gamma }\lambda_{\gamma }$$ (2)

where the empirical estimate from the data $\hat{E}{\left(X_{\gamma }\lambda_{\gamma }\right)}^{2}$ equals zero because of centered genotypes. Hence our point estimate is $E({\hat{h}}_{\gamma }^{2})=\mu_{\gamma }^{T}{\hat{R}}_{\gamma }\mu_{\gamma }+tr({\hat{R}}_{\gamma }\Sigma_{\gamma })$ and we compute the posterior distribution of $h_{\gamma }^{2}$ by evaluating Eq (2) for each draw from the posterior for ***λ***_*γ*_. This approach allows us to obtain the mean, standard deviation and 95% credible interval (equal tail area interval) of the posterior. We expect high quality posterior inference if ${\hat{R}}_{\gamma }$ is a good approximation to the pairwise sample correlations computed from the original GWAS genotype data [24].

### Large effect sizes

For quantitative traits, setting the estimates for residual variances in $\hat{D}$ equal to one is a good approximation in genomic regions with small causal effects and FINEMAP v1.1 uses this approximation. However, this approach can lead to problems when causal variants have larger effect sizes [25]. Since FINEMAP v1.2, we account for different magnitudes of effect sizes by calculating, for each variant ℓ, the maximum likelihood estimate

$${\hat{\sigma }}_{l}^{2}={\left(1+{\hat{z}}_{l}^{2}/n\right)}^{-1}$$

for the residual variance in univariate linear regression. If all variants individually account for less than heritability of 1%, we keep ${\hat{\sigma }}_{\gamma }^{2}$ in Eq (1) fixed at a value of 1.0 and set the prior variance for the effect size $s_{\lambda }^{2}$ equal to 0.05^2^; this means that a causal variant contributes less than heritability of 1% with probability 0.95 [18]. Otherwise, we expect to see larger effect sizes. In this case, we set ${\hat{\sigma }}_{\gamma }^{2}$ in Eq (1) equal to the maximum likelihood estimate for the residual variance from multiple linear regression

$${\hat{\sigma }}_{\gamma }^{2}=1-\frac{{\hat{z}}_{\gamma }^{T}{\hat{D}}_{\gamma }^{1/2}{\hat{R}}_{\gamma }^{-1}{\hat{D}}_{\gamma }^{1/2}{\hat{z}}_{\gamma }}{n}$$

of variants in the causal configuration *γ*. For each causal configuration, we further use an equidistant grid of four values for the prior variance $s_{\lambda }^{2}$ with a lower bound at 0.05^2^ and an upper bound where, with probability 0.95, a causal variant contributes less than the point estimate of heritability ${\hat{h}}_{\gamma }^{2}=1-{\hat{\sigma }}_{\gamma }^{2}$ of a causal configuration *γ* of the variants with pairwise absolute correlations less than 0.95 that are marginally the most significant and that each contribute more than heritability of 1%. For each grid value, we compute the Bayes factor for assessing the evidence against the null configuration. We obtain the final Bayes factor by computing the average value of the individual Bayes factors. This model averaging corresponds to a uniform prior distribution on $s_{\lambda }^{2}$ over the grid points.

Our strategy for adapting to large effect sizes requires no actions from the user and therefore differs from the approach [25] of CAVIARBF v0.2.1 (see Web Resources) which requires user-given values for the prior standard deviation *s*_*λ*_. We compare FINEMAP v1.4 with CAVIARBF and FINEMAP v1.1 on simulated datasets with three causal variants among 150 variants in a genomic region (see “Simulations with GWAS regions”). Each dataset was analyzed with FINEMAP and CAVIARBF using default values and allowing for a maximum of four causal variants in the region. For CAVIARBF, we excluded the null configuration from the output and used multiple values 0.05, 0.1, 0.2, 0.4, 0.8 to specify the prior standard deviation *s*_*λ*_ as recommended [25].

### Bayesian model-averaging

The Bayesian variable selection approach to fine-mapping causal variants is a hard combinatorial problem which precludes exhaustive enumeration of all possible causal configurations. In previous work [18], we tackled the combinatorial problem with Shotgun Stochastic Search [26] algorithm to identify a list of causal configurations which captures a large majority of the total posterior probability [18]. According to the Bayesian paradigm, we account for model uncertainty through Bayesian Model Averaging (BMA) [27]. BMA combines inference about a quantity common to all models by averaging the posterior distribution of that quantity under each model using posterior model probabilities as weights.

Using BMA, we compute the PIP for the ℓth variant by summing up the posterior probability of all causal configurations in which this variant is included. We model-average estimates of heritability to quantify the contribution of causal variants. Examples of other useful model-averaged estimates are the posterior effect size mean and standard deviation of the ℓth variant. We output marginalized shrinkage estimates of the posterior effect size mean and standard deviation by averaging over all causal configurations assuming that the effect size of the ℓth variant is zero if the variant is absent from a causal configuration. We also output the conditional estimates of the posterior effect size mean and standard deviation conditional on inclusion by averaging over only those causal configurations in which the ℓth variant is included. The magnitude of the conditional model-averaged estimate of the ℓth variant is much larger than the shrinkage model-averaged estimate if the PIP for the ℓth variant is small.

### Polygenic and fixed-effect models

Variance component models are extensively used for heritability estimation under a polygenic model assumption. We performed regional heritability estimation from individual-level genotype-phenotype data with BOLT v2.3 (see Web Resources) using default settings. In genome-wide analyses, we used an LD pruned set of variants (—indep-pairwise 1000 100 0.8, see Web Resources) while in regional analyses we used an unpruned set of variants to maintain comparability to other methods that estimate regional heritability.

Since the model assumption of polygenicity may not hold in any particular genomic region [14], we also estimated regional heritability with HESS v0.5.2 (see Web Resources) to avoid assumptions about causal effect sizes. HESS requires GWAS summary statistics and genotype data to compute pairwise correlations between the variants to account for LD. To keep the allele coding between GWAS summary statistics and pairwise correlations consistent and maintain comparability with FINEMAP, we modified HESS to read in the same pre-computed pairwise correlations obtained from the original GWAS genotype data as we use in FINEMAP. We further used marginal effect size estimates on the standardized scale as input to HESS (personal communication with the authors of HESS). We ran HESS on approximately independent LD blocks [28] using all variants in the genomic region and default settings. We also ran HESS without default adjustment for noise in the LD matrix by setting the command-line flags—max-num-eig *m* and --min-eigval 10^−8^, as recommended by the authors of HESS, where *m* is the number of variants in the genomic region.

## Genotype data

We used genotype data on 426,844 individuals of EUR ancestry (see Web Resources) provided by UKB. For documentation about imputation of the UKB genotype data see Web Resources. We retained genotyped biallelic variants with MAF greater than 1% and excluded related individuals with KING’s [29] kinship coefficient greater than 0.0442 using a greedy algorithm that maximizes the final sample size. This left us with 374,073 samples in UKB.

### Phenotype data

We used plasma level concentrations of 2,940 unique proteins measured with the antibody-based Olink Explore 3072 Proximity Extension Assay (PEA) across a randomly chosen set of 32,867 unrelated individuals of European ancestry included in UKB. Details about the proteomics data are given in Sun et al. [30]. Protein level concentrations were adjusted by age, age^2^, sex, age × sex, age^2^ × sex, batch, UKB assessment center, UKB genetic array, time between blood sampling and measurement and the first 20 genetic principal components using a linear regression model. Regression residuals were normalized using a rank-based transformation to the standard Normal distribution.

### Protein-associated genomic regions

The UK Biobank Pharma Proteomics Projects (UKB-PPP) used REGENIE (see Web Resources) to test genome-wide associations with 2,940 proteins. We defined the level of statistical significance as P < 1.7×10^−11^ corresponding to the standard genome-wide significance level (P < 5 × 10^−8^) corrected for the number of proteins analyzed (5 × 10^−8^ / 2940 = 1.7×10^−11^). The pQTLs were defined through clumping ±1Mb around statistically significant variants, excluding the human leukocyte antigen (HLA) region which is treated as one locus due to complex and extensive LD patterns. Physically overlapping regions were merged into one, deeming the variant with the lowest P-value as the lead variant.

We estimated regional heritability with BOLT, HESS and FINEMAP across 14,189 pQTLs from the UKB-PPP in 28,008 unrelated individuals of European ancestry. Our motivation to use unrelated individuals is to prevent the possibility of bias across the three methods. For FINEMAP and HESS we generated summary statistics with a linear model implemented in PLINK2 (see Web Resources). With FINEMAP we either allowed for thirty causal variants or only one causal variant to estimate the heritability captured by the lead variant. The polygenic component of heritability was estimated with BOLT using genome-wide variants outside of regions defined by pQTLs ±5Mb. In the Paraoxonase 1 *cis*-pQTL, we performed a stepwise conditional analysis implemented in PLINK2 by first conditioning on the variant with the lowest P-value, hereafter called the lead variant, and then iteratively adding to the model the variant with the lowest conditional P-value until no further variant reached the standard genome-wide significance threshold (P < 5 × 10^−8^) or until we had conducted a pre-specified number of iterations.

### Simulations with GWAS regions

To assess how different methods estimate regional heritability we performed comprehensive simulations over genomic regions chosen from GWAS meta-analyses for coronary artery disease (CAD) [31], Crohn’s disease (CD) [32], lipid traits (LIPs) [33], schizophrenia (SCZ) [34], and type 2 diabetes (T2D) [35]. For each study, we retained the lead variants outside the HLA region with a marginal P-value less than 5 × 10^−8^ and selected 100 lead variants (18 from CAD, 20 from CD, 21 from LIPs, 21 from SCZ, and 20 from T2D) for further analyses. Around each lead variant, we defined genomic regions of 3 Mb. We considered the following fine-mapping setting:

- **Scenario H**

Increasing regional heritability (*h*^*2*^ = 0.00025, 0.0015, 0.05, 0.5)

- **Scenario M**

Increasing number of causal variants (*m* = 3, 50)

- **Scenario N**

Increasing GWAS sample size (*n* = 5,000, 50,000, 300,000)

A total of 1,000 datasets (10 replicates per each region) were generated under each of the 24 combinations of scenarios H, M and N using the following linear model

$$y=\sum_{c\in C}\beta_{c}g_{c}+\epsilon$$

where *C* is the set of causal variants, ***g***_*c*_ the vector of standardized genotypes from 374,073 UKB individuals at the *c*th causal variant, *β*_*c*_ the standardized effect size of the *c*th variant and ***ϵ*** is Normal noise with mean 0 and variance $\sigma_{\epsilon }^{2}=(1-h^{2})$.

Causal variants were randomly chosen. In datasets with three causal variants, the joint causal effect sizes were specified such that in the true causal configuration, the three variants accounted for 61.8%, 25.8% and 12.4% of the regional heritability, respectively (That is, the effect size of a standardized genotype variable of a causal variant was (*h*^2^×*p*)^1/2^, where *h*^2^ is the regional heritability and *p* is the proportion of heritability accounted for by the variant). These per-variant proportions were chosen from a meta-analysis of the association between *MC4R* locus and body mass index [36]. In simulations with fifty causal variants, joint causal effect sizes were specified such that each causal variant accounted for the same proportion of regional heritability.

For each set of 1,000 simulations, we report the average heritability estimates and the average standard error (BOLT, HESS) or posterior standard deviation (FINEMAP). We also assess the uncertainty of heritability estimates across 100 simulated datasets of the same region with causal variants kept fixed. For 95% credible and confidence intervals (normal approximations), we calculate their coverage as the proportion of intervals that covered the true level of heritability. We also compute the RMSE defined as the square root of the average squared deviation of the estimated heritability from the true level of heritability. RMSE summarizes both bias and precision of the estimation procedures with smaller RMSE values indicating better performance.

## Web Resources

- **BOLT**

Loh PR, Tucker G, Bulik-Sullivan BK, Vilhjálmsson BJ, Finucane HK, Salem RM, et al. (2015). Efficient Bayesian mixed-model analysis increases association power in large cohorts. Nat. Genet. 47, 284–290. *10.1038/ng.3190*. Available from: https://data.broadinstitute.org/alkesgroup/BOLT-LMM

- **CAVIARBF**

Chen W, Larrabee BR, Ovsyannikova IG, Kennedy RB, Haralambieva IH, Poland GA, et al. (2015). Fine Mapping Causal Variants with an Approximate Bayesian Method Using Marginal Test Statistics. Genetics 200, 719–736. 10.1534/genetics.115.176107. Available from: https://bitbucket.org/Wenan/caviarbf

- **FINEMAP**

Benner C, Spencer CCA, Havulinna AS, Salomaa V, Ripatti S, Pirinen M (2016). FINEMAP: efficient variable selection using summary data from genome-wide association studies. Bioinformatics 32, 1493–1501. 10.1093/bioinformatics/btw018. Available from: http://www.finemap.me

- **HESS**

Shi H, Kichaev G, Pasaniuc B (2016). Contrasting the Genetic Architecture of 30 Complex Traits from Summary Association Data. Am. J. Hum. Genet. *99, 139–153. 10.1016/j.ajhg.2016.05.013. Available from: https://github.com/huwenboshi/hess*

- **PLINK**

Chang CC, Chow CC, Tellier LCAM, Vattikuti S, Purcell SM, Lee JJ (2015) Second-generation PLINK: rising to the challenge of larger and richer datasets. GigaScience, 4. Available from: https://www.cog-genomics.org/plink

- **REGENIE**

Mbatchou J, Barnard L, Backman J, Marcketta A, Kosmicki JA, Ziyatdinov A, et al. (2021) Computationally efficient whole-genome regression for quantitative and binary traits. Nat. Genet. 53, 1097–1103. 10.1038/s41588-021-00870-7. Available from: https://rgcgithub.github.io/regenie/

- **UKB imputation**

Marchini J. UK Biobank Phasing and Imputation Documentation 2015 May 13 [cited 23 December 2024]. UK Biobank website [Internet]. Available from: http://www.ukbiobank.co.uk/wp-content/uploads/2014/04/imputation_documentation_May2015.pdf

## Supporting information

S1 Table Marginal and conditional P-values from the PON1 GWAS for the *cis*-pQTL with 27,607 individuals from UKB. (DOCX)

S2 Table (A) Average heritability estimates and their uncertainty from FINEMAP, BOLT and HESS in simulations over GWAS regions with three randomly chosen causal SNPs. (B) Uncertainty quantification for FINEMAP, BOLT and HESS in simulations over a single GWAS region with three randomly chosen causal SNPs kept fixed.
(DOCX)

S3 Table (A) Average heritability estimates and their uncertainty from FINEMAP, BOLT and HESS in simulations over GWAS regions with fifty randomly chosen causal SNPs. (B) Uncertainty quantification for FINEMAP, BOLT and HESS in simulations over a single GWAS region with fifty randomly chosen causal SNPs kept fixed.
(DOCX)

S4 Table Region-specific results of 14,189 pQTLs. (XLSX)

S1 Fig Genotype data from UKB for 150 variants were used for phenotype generation. Three GWAS sample sizes (*n*) and three heritability values (*h*^2^) were considered and for each combination of sample size and heritability, 10 datasets were generated. Each dataset included three causal variants with joint effect sizes chosen so that the three variants together account for the regional heritability in proportions of 61.8%, 25.8% and 12.4%. CAVIARBF and FINEMAP were run with default settings allowing for 4 causal variants. CAVIARBF used 0.05, 0.1, 0.2, 0.4, 0.8 as prior standard deviation s_λ_, whereas FINEMAP v1.1 used s_λ_ = 0.05. Panels compare posterior inclusion probabilities from FINEMAP v1.4 with posterior inclusion probabilities from CAVIARBF (A) or FINEMAP v1.1 (B). Causal variants are highlighted as purple squares and the other variants as dark khaki dots.
(TIFF)

S2 Fig Distribution of the posterior expectation of the number of causal variants from FINEMAP across simulations over 100 GWAS regions. Three GWAS sample sizes (*n*), four heritability values (*h*^2^) and two genetic architectures (*m*) were considered and for each combination of sample size, heritability and genetic architecture, 10 datasets were generated per GWAS region. Datasets with sparse genetic architecture included three causal variants with joint effect sizes chosen so that the three variants together account for the regional heritability in proportions of 61.8%, 25.8% and 12.4%. Datasets with polygenic genetic architecture included fifty causal variants with joint causal effect sizes specified in such a way that each causal variant accounts for the same proportion of regional heritability. FINEMAP was run with default settings allowing for 100 causal variants.
(TIFF)
