# Supplementary material for: Refining fine-mapping: Effect sizes and regional heritability
Source: PLoS Genet. 2025 Jan 9;21(1):e1011480. doi: 10.1371/journal.pgen.1011480 (PMC11753704; doi:10.1371/journal.pgen.1011480)
Supplement: S1 Table — (DOCX) [file pgen.1011480.s001.docx]

| **S1 Table** | **Marginal and conditional P-values from the PON1 GWAS for the *cis*-pQTL with 27,607 individuals from UKB** | | |
| --- | --- | --- | --- |
| Iteration | Variant | Marginal P- value | P-value after conditioning on all variants from previous rounds |
| 1 | rs854560 | 5.8×10^-1466^ |  |
| 2 | rs7493 | 1.8×10^-920^ | 2.3×10^-621^ |
| 3 | rs3917510 | 3.0×10^-35^ | 2.1×10^-266^ |
| 4 | rs662 | 1.2×10^-1042^ | 5.3×10^-229^ |
| 5 | rs3917478 | 1.3×10^-244^ | 2.1×10^-172^ |
| 6 | rs2240026 | 5.4×10^-1^ | 6.9×10^-129^ |
| 7 | rs17883513 | 1.5×10^-423^ | 2.0×10^-73^ |
| 8 | rs112854772 | 1.7×10^-6^ | 5.2×10^-45^ |
| 9 | rs17886762 | 2.8×10^-271^ | 4.7×10^-32^ |
| 10 | rs17305704 | 7.1×10^-202^ | 3.3×10^-41^ |
| 11 | rs13226149 | 1.0×10^-941^ | 2.3×10^-21^ |
| 12 | rs854548 | 2.5×10^-546^ | 6.2×10^-22^ |
| 13 | rs854555 | 1.1×10^-424^ | 2.5×10^-99^ |
| 14 | rs7797678 | 5.1×10^-3^ | 2.1×10^-12^ |
| 15 | rs7802018 | 2.8×10^-271^ | 7.5×10^-11^ |
| 16 | rs43053 | 4.1×10^-178^ | 1.7×10^-15^ |
| 17 | rs2374993 | 2.3×10^-94^ | 1.4×10^-14^ |
| 18 | rs7803148 | 3.0×10^-774^ | 6.5×10^-14^ |
| 19 | rs112592935 | 3.1×10^-3^ | 1.2×10^-15^ |
| 20 | rs73235074 | 1.3×10^-28^ | 4.8×10^-10^ |
