# Supplementary material for: Refining fine-mapping: Effect sizes and regional heritability
Source: PLoS Genet. 2025 Jan 9;21(1):e1011480. doi: 10.1371/journal.pgen.1011480 (PMC11753704; doi:10.1371/journal.pgen.1011480)
Supplement: S3 Table — (A) Average heritability estimates and their uncertainty from FINEMAP, BOLT and HESS in simulations over GWAS regions with fifty randomly chosen causal SNPs. (B) Uncertainty quantification for FINEMAP, BOLT and HESS in simulations over a single GWAS region with fifty randomly chosen causal SNPs kept fixed. (DOCX) [file pgen.1011480.s003.docx]

| **S3A Table** | **Average heritability estimates and their uncertainty from FINEMAP, BOLT and HESS in simulations over GWAS regions with fifty randomly chosen causal SNPs** | | | | | | | | | | | |
| --- | --- | --- | --- | --- | --- | --- | --- | --- | --- | --- | --- | --- |
|  | |  |  | Average h2 estimates | | | |  | Average SE | | | |
| Sample size | | True h2 |  | FINEMAP | BOLT | HESS with regularization | HESS without regularization |  | FINEMAP | BOLT | HESS with regularization | HESS without regularization |
| 5000 | | 0.00025 |  | 0.00101 | 0.00236 | 0.00022 | 0.00046 |  | 0.00073 | 0.00411 | 0.00767 | 0.00766 |
|  |  | 0.00150 |  | 0.00116 | 0.00329 | 0.00118 | 0.00178 |  | 0.00078 | 0.00419 | 0.00772 | 0.00773 |
|  |  | 0.05000 |  | 0.02591 | 0.04856 | 0.04154 | 0.06225 |  | 0.00423 | 0.00933 | 0.00949 | 0.01019 |
|  |  | 0.50000 |  | 0.48136 | 0.44451 | 0.41535 | 0.62283 |  | 0.01389 | 0.02193 | 0.01529 | 0.01469 |
| 50000 | | 0.00025 |  | 0.00015 | 0.00046 | 0.00020 | 0.00030 |  | 0.00010 | 0.00044 | 0.00074 | 0.00074 |
|  |  | 0.00150 |  | 0.00052 | 0.00166 | 0.00123 | 0.00172 |  | 0.00019 | 0.00061 | 0.00079 | 0.00082 |
|  |  | 0.05000 |  | 0.04748 | 0.04104 | 0.04108 | 0.05620 |  | 0.00189 | 0.00374 | 0.00191 | 0.00218 |
|  |  | 0.50000 |  | 0.49052 | 0.45505 | 0.40990 | 0.56154 |  | 0.00446 | 0.01577 | 0.00439 | 0.00445 |
| 300000 | | 0.00025 |  | 0.00008 | 0.00027 | 0.00020 | 0.00028 |  | 0.00003 | 0.00010 | 0.00013 | 0.00013 |
|  |  | 0.00150 |  | 0.00098 | 0.00137 | 0.00123 | 0.00167 |  | 0.00011 | 0.00022 | 0.00018 | 0.00019 |
|  |  | 0.05000 |  | 0.04980 | 0.04147 | 0.04085 | 0.05560 |  | 0.00079 | 0.00279 | 0.00073 | 0.00084 |
|  |  | 0.50000 |  | 0.49048 | 0.46841 | 0.40792 | 0.55699 |  | 0.00182 | 0.01454 | 0.00177 | 0.00181 |

| **S3B Table** | **Uncertainty quantification for FINEMAP, BOLT and HESS in simulations over a single GWAS region with fifty randomly chosen causal SNPs kept fixed** | | | | | | | | | | | | | |
| --- | --- | --- | --- | --- | --- | --- | --- | --- | --- | --- | --- | --- | --- | --- |
|  |  |  | Average SE / SD of h2 estimates | | | | Coverage | | | | Root Mean Square Error | | | |
| Sample size | True h2 |  | FINEMAP | BOLT | HESS | | FINEMAP | BOLT | HESS | | FINEMAP | BOLT | HESS | |
|  |  |  |  |  | With regular-ization | Without regular-ization |  |  | With regular-ization | Without regular-ization |  |  | With regular-ization | Without regular-ization |
| 5000 | 0.00025 |  | 1.434 | 1.348 | 2.594 | 0.786 | 0.840 | 0.59 | 1.00 | 0.89 | 0.00091 | 0.00419 | 0.00312 | 0.01024 |
|  | 0.00150 |  | 1.010 | 1.257 | 2.596 | 0.788 | 0.990 | 0.72 | 1.00 | 0.87 | 0.00084 | 0.00454 | 0.00314 | 0.01029 |
|  | 0.05000 |  | 0.717 | 1.312 | 1.584 | 0.863 | 0.000 | 0.99 | 0.90 | 0.78 | 0.02809 | 0.00721 | 0.01253 | 0.01761 |
|  | 0.50000 |  | 1.621 | 1.547 | 1.211 | 0.666 | 0.000 | 0.94 | 0.00 | 0.00 | 0.05264 | 0.02152 | 0.13356 | 0.12610 |
| 50000 | 0.00025 |  | 1.257 | 1.244 | 2.205 | 0.904 | 0.930 | 0.74 | 1.00 | 0.91 | 0.00014 | 0.00041 | 0.00036 | 0.00086 |
|  | 0.00150 |  | 0.745 | 1.321 | 2.116 | 1.083 | 0.080 | 1.00 | 1.00 | 0.95 | 0.00104 | 0.00048 | 0.00051 | 0.00078 |
|  | 0.05000 |  | 1.042 | 1.439 | 1.088 | 0.920 | 0.280 | 1.00 | 0.00 | 0.68 | 0.00502 | 0.00289 | 0.01853 | 0.00386 |
|  | 0.50000 |  | 0.346 | 2.264 | 1.146 | 0.900 | 0.000 | 1.00 | 0.00 | 0.00 | 0.05741 | 0.01258 | 0.14705 | 0.04217 |
| 300000 | 0.00025 |  | 0.832 | 1.162 | 1.993 | 1.031 | 0.020 | 0.96 | 1.00 | 0.95 | 0.00020 | 0.00009 | 0.00008 | 0.00014 |
|  | 0.00150 |  | 0.584 | 1.284 | 1.177 | 0.926 | 0.090 | 0.97 | 0.33 | 0.91 | 0.00057 | 0.00019 | 0.00045 | 0.00022 |
|  | 0.05000 |  | 1.132 | 2.149 | 1.034 | 0.961 | 0.000 | 1.00 | 0.00 | 0.00 | 0.00476 | 0.00260 | 0.01397 | 0.00534 |
|  | 0.50000 |  | 0.041 | 4.315 | 1.199 | 0.968 | 0.000 | 0.51 | 0.00 | 0.00 | 0.07951 | 0.02679 | 0.14189 | 0.03837 |
