# Supplementary figures and images for: Refining fine-mapping: Effect sizes and regional heritability
Source: PLoS Genet. 2025 Jan 9;21(1):e1011480. doi: 10.1371/journal.pgen.1011480 (PMC11753704; doi:10.1371/journal.pgen.1011480)

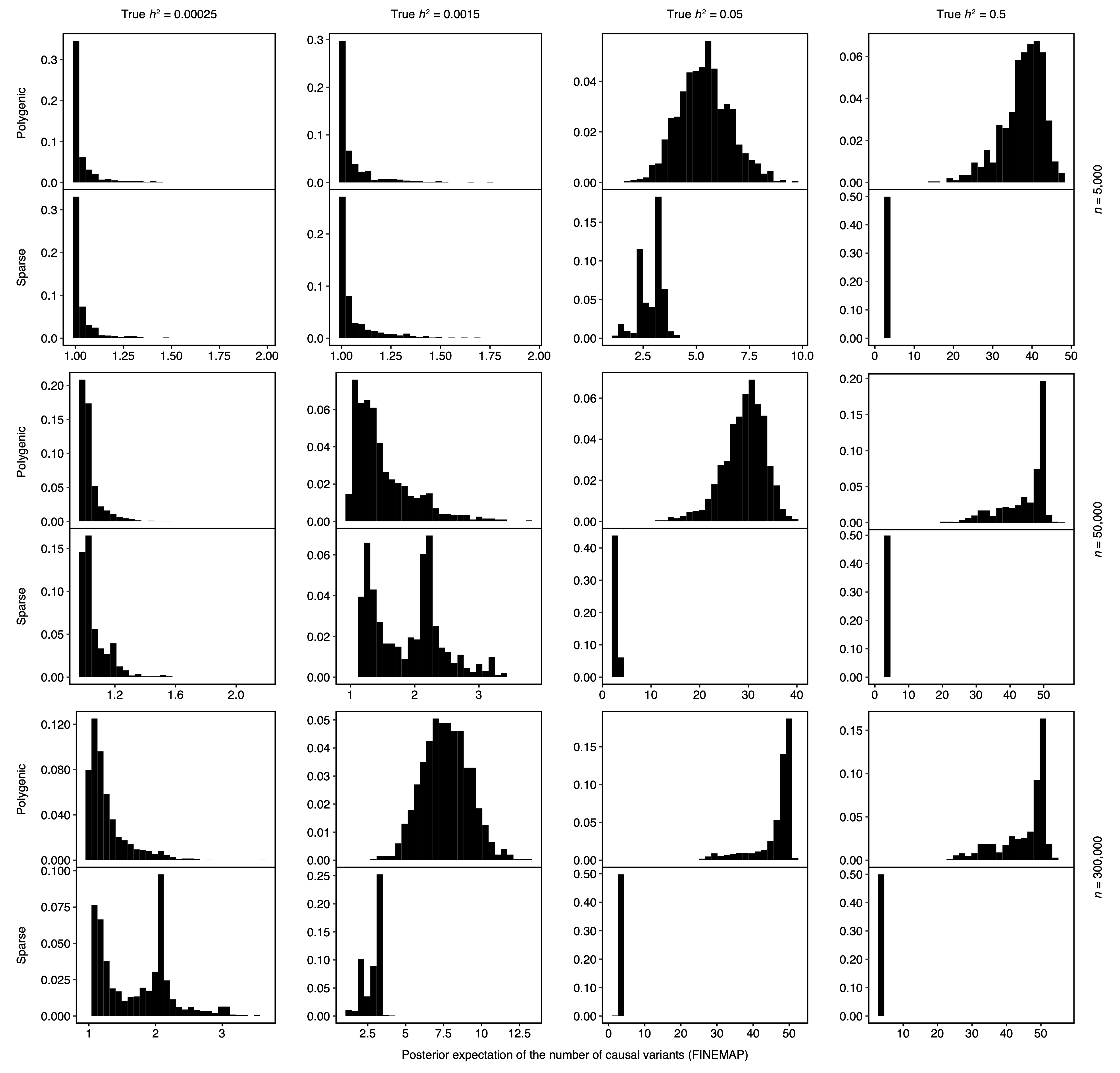

Supplement: S2 Fig — Three GWAS sample sizes (n), four heritability values (h2) and two genetic architectures (m) were considered and for each combination of sample size, heritability and genetic architecture, 10 datasets were generated per GWAS region. Datasets with sparse genetic architecture included three causal variants with joint effect sizes chosen so that the three variants together account for the regional heritability in proportions of 61.8%, 25.8% and 12.4%. Datasets with polygenic genetic architecture included fifty causal variants with joint causal effect sizes specified in such a way that each causal variant accounts for the same proportion of regional heritability. FINEMAP was run with default settings allowing for 100 causal variants. (TIFF) [file pgen.1011480.s006.tiff]
